# 24-Month Effectiveness of Periduoscopic Adhesiolysis in Reducing the Use of Spinal Cord Stimulation in Patient With Chronic Lumbar Pain: A Possible Therapeutic Regimen?

**DOI:** 10.7759/cureus.17563

**Published:** 2021-08-30

**Authors:** Maurizio Marchesini, Marco Baciarello, Roberto Bellacicco, Edoardo Flaviano, Elena G Bignami

**Affiliations:** 1 Anesthesia and Critical Care, Instituti Clinici Scientifici (ICS) Maugeri, Pavia, ITA; 2 II Service Anesthesia, Critical Care and Pain Medicine, Azienda Ospedaliero Universitaria Parma, Parma, ITA; 3 II Service Anesthesia, Critical Care and Pain Medicine, Azienda Ospedaliero Univeristaria Parma, Parma, ITA; 4 Anesthesia and Critical Care, Papa Giovanni XXIIII, Bergamo, ITA; 5 II Service Anesthesia, Critical Care and Pain Medicine, Azienda Ospedaliera Universitaria Parma, Parma, ITA

**Keywords:** periduroscopy, spinal cord stimulation, chronic pain, hta, chronic post surgical pain

## Abstract

Objectives

Epiduroscopy is a minimally invasive procedure that is used in pain therapy to treat lumbar and root pain that is resistant to medical and infiltrative therapies. The indications for periduroscopy are partly shared with those of spinal cord stimulation (SCS): failed back surgery syndrome (FBSS) and stenosis of the vertebral canal in particular. The costs and risks of periduroscopy are considerably lower than those of SCS. The purpose of this study is to evaluate the clinical and economic advantages of integrating periduroscopy as a step prior to SCS for patients with severe lumbar or radicular pain that is unresponsive to pharmacological and infiltrative treatments.

Materials and Methods

Patients were enrolled if they had FBSS and spinal stenosis with indications for SCS and accepted periduroscopy treatment before the possible SCS trial. Patients were followed up for 24 months with evaluations of clinical data on the day after the procedure and at one and 24 months. The pain trend, satisfaction with the periduroscopy procedure, and the incidence of SCS implants in the study period were analyzed.

Results

A total of 106 patients were enrolled. Immediately after the procedure and in the first month, the reduction of pain and the level of patient satisfaction were high, but they were drastically reduced at 24 months with a progressive reappearance of symptoms that substantially overlapped with the pre-surgery levels. At 24 months, 48% of the patients underwent a neurostimulation trial, and a significant percentage of them were able to avoid the implantation of an SCS.

Conclusions

Periduroscopy appears to be rational as a step prior to SCS in terms of the improvement of pain symptoms in the short term, the definitive results in a significant percentage of patients, and the significant economic savings for the health system.

## Introduction

The story of epiduroscopy is one of intuition [1]. It is the same kind of intuition that is shared with the great seafarers who profoundly changed our way of thinking about the world in the XV century and moved the borders of the known universe a bit forward. But in this case, the "New World" is the features of the tiny space known as the epidural space. But this anatomical space has been well known over the centuries, so the attribute “new” is not suitable. What is really “new” is the insight that allows us to think of it as navigable and explorable, regardless of the size (the normal diameter ranges are between 15 and 27 mm for the vertebral canal and between 20 and 23 mm for the intervertebral foramen) [2].

The first endoscopic study of the spinal canal was made using arthroscopic equipment and vertebral columns removed from a cadaver, dating back to Burman [3] in the early 1930s. After the first reports, the technique was no longer mentioned for decades until 1967 [4]. Only by the end of 1980 has it been primarily used for examining the epidural space thanks to the development of small-caliber flexible optics [5]. Since then, many other improvements have been made, and the interest has never receded; at last, the power of that original intuition came across to technological progress.

Epiduroscopy is a minimally invasive technique that is useful for assessing the epidural space through a flexible instrument connected to an optical fiber. In addition to simple clinical evaluation, epiduroscopy allows for treatments such as the targeted delivery of drugs, debridement of adhesions, and reduction of inflammatory factors with copious lavage [6-7]. Even in non-operated patients (so-called "virgin back"), epiduroscopy can be a valuable approach for possible interventions on anatomical structures that can be pain generators in pathological conditions [8]. However, "minimally invasive" does not mean "free of complications." Transient neurological symptoms (TNS) and post-procedural pain in the affected area are the most frequent and benign manifestations. Loss of acuity, encephalopathy resulting in rhabdomyolysis, neurological bladder, seizures, and pneumocephalus are among the other complications worth mentioning because of their potential severe and permanent consequences, although they are much less common [9].

Spinal cord stimulation (SCS) was first described and applied by Shealy [10] in the late 1960s and has become a widely used and effective alternative for the management of chronic refractory pain that is unresponsive to conservative therapies [11-13] even in elderly patients [14]. Although the clinical benefit of SCS is substantial, detailed knowledge of how SCS inhibits pain is lacking. The technique acts both at the spinal [15] (mostly) and the supraspinal [16] levels, depending on the type of stimulation pattern. It is believed to work according to gate control theory [17] by stimulating the large-size axons (A-beta fibers) [18] and interneurons in the dorsal roots, thus decreasing the influx of painful stimuli.

SCS may evoke the release of serotonin and norepinephrine into the dorsal horn from descending fibers originating in the supraspinal pain-modulatory structures [19]. Other changes in the neurochemical environment at the spinal level are related to the local concentrations of gamma-aminobutyric acid (GABA) and acetylcholine (ACh) [20]. Interestingly, this technology does not only have immediate action; its positive effects have been revealed to be long-lasting because of neuronal plasticity [21].

The most common complication in SCS is hardware problems, which include electrode migrations and lead fracture [22]. The most significant complications are associated with neurological damage due to intraoperative root or spinal cord injury, epidural hematoma, and infections (particularly epidural abscess) [23]. Notably painful stimulation and inadequate coverage of the painful area sometimes necessitate either repositioning or removing the electrode [24]. In 1998, it was estimated that SCS was applied to about 15 000 patients annually worldwide, of which about 5000 were cases in Europe [25]. Nowadays, the number has at least doubled.

According to the "Pain in Europe" study [26], up to 19% of the whole European population suffers from moderate or severe back pain. In Italy. this percentage is even higher reaching 26%. Back pain has profound consequences on people's lives and incurs tremendous costs for society since most individuals are working age. What we are facing here is a healthcare emergency, and the right tool needs to be used for the right candidate to avoid further waste of time and economic resources.

The aim of this study is to determine whether epiduroscopy is capable of playing a significant role in the treatment regimen of severe lumbar pain that does not respond to standard medical therapies.

## Materials and methods

This study was approved by the responsible ethics committee, and informed consent was acquired from all patients. We enrolled 106 patients with failed back surgery syndrome (FBSS) and stenosis of the canal. Patients were enrolled with the following inclusion criteria:

- Age 18-85 years

- Patients with FBSS and spinal stenosis

- Able to sign an informed consent form

The following exclusion criteria were applied:

- Patients with clinically unstable disease (all morbid forms whose treatment is not stable over time but requires continuous pharmacological and dosage adjustments or require further investigations)

- Patients who have been diagnosed with severe neuropsychiatric disease and have received pharmacological treatment under constant medical supervision for which there is a contraindication for invasive treatments

- History of vertebral fractures

- Tumors or infections affecting the spine

- Visual impairment (glaucoma, diabetic retinopathy)

- Chronic primary or secondary headache

- Pregnancy

- Coagulopathies (INR > 1. 5)

Analgesic visit and follow-up

The patients underwent an antalgic visit before admission with an evaluation of the blood count, coagulation, ECG, and anesthesiological examination. Pain characteristics were assessed before the periduroscopic procedure, immediately after the procedure, at one month, and at 24 months using a questionnaire, in which the Numeric Rating Scale (NRS) and Global Perceived Effect (GPE) indices were analyzed. The NRS value was calculated by asking the patients about their minimum, average, and maximum intensity of pain during the day on a numerical scale of one to 10. The GPE value was calculated by asking patients to rate their pain relief compared to baseline as > 50%, between 30 and 50%, and < 30%. The costs incurred by the National Health Service (NHS) for the periduroscopic adhesiolysis procedure (LOA) and SCS were analyzed (costs in 2016-2018).

Periduroscopy

All the procedures were performed by a single operator who had experience with more than 80 procedures. The procedures were performed with the Resascope System (AMS group srl, Padua, Italy), which is composed of a 10F adjustable epidural video-guided catheter with an external diameter of 3.3 mm, a length of 30 cm, and two channels. One channel is for saline infusion for washing the epidural space, thus improving visibility, and the other one is for the collection of the fluid. Three entry channels are used for the insertion of flexible fiber optics and other tools and have an internal diameter of 1.25 mm.

## Results

Demographics

We recruited 106 patients (41 men and 65 women) who underwent periduroscopic adhesiolysis and were diagnosed with FBSS (92 pcs) or canal stenosis (14 pcs). The median age was 57 years (range 30-80 years).

Adverse events, screening failure, and dropout

**Figure 1:**
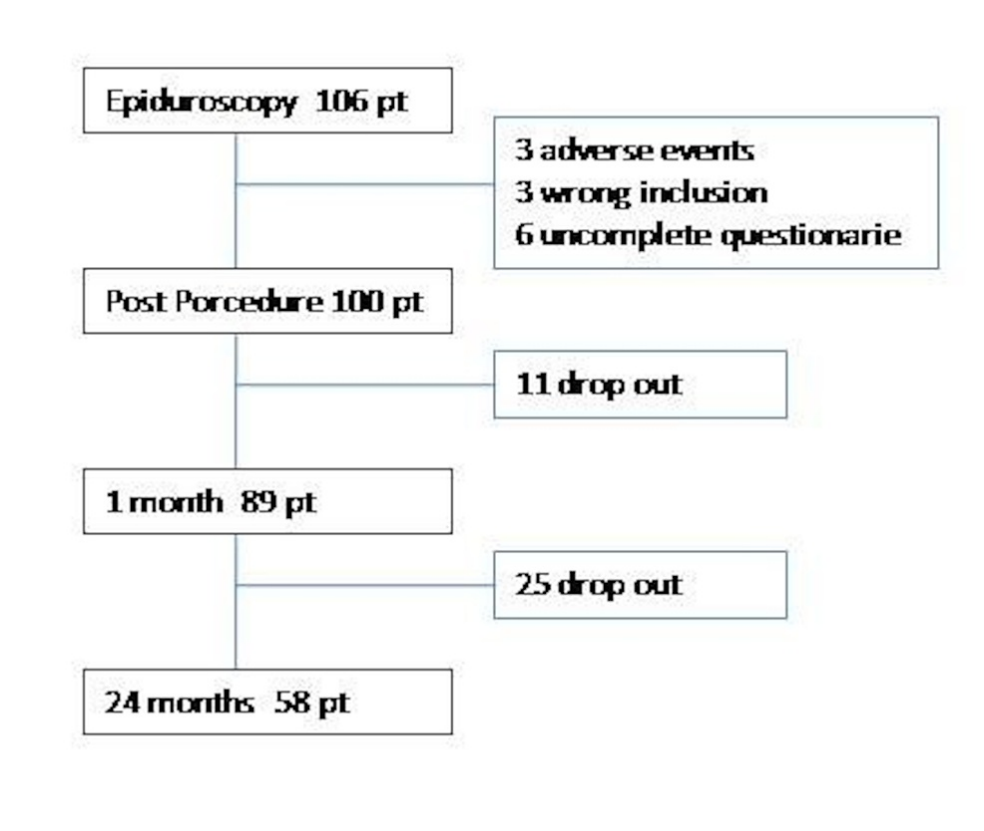
Patients' screening

During the procedure, three adverse events that led to stopping the procedure were recorded: retinal hemorrhage, significant peridural bleeding, and hemodynamic instability. During the first month, three patients were excluded from the study due to no longer fulfilling the inclusion and exclusion criteria. It was not possible to administer the questionnaire to six patients after the procedure, to 11 patients at the follow-up at one month, and to 25 patients at the follow-up at 24 months. Thus, there were 58 patients in the study at 24 months (Figure 1).

NRS and GPE evaluation

The pain tendencies were similar for the maximum, average, and minimum NRS. There was a significant reduction after the procedure, a relative rise at one month, and a substantial return to near the baseline value at 24 months. The values and statistical analysis results are summarized in Table 1.

**Table 1:** Pain relief after periduroscopy NRS: Numeric Pain Rating Scale

	Baseline	After procedure	1 month	24 months	Baseline vs. after procedure	Baseline vs. 1 month	Baseline vs. 24 months
NRS Max	8.8	4.2	6.2	7.2	p < 0.001	p < 0.01	n. s.
NRS Average	6.7	3.9	5.4	6.5	p < 0.001	p < 0.01	n. s.
NRS Min	3	1.2	2.1	2.4	p < 0.001	n. s.	n. s.

**Figure 2:**
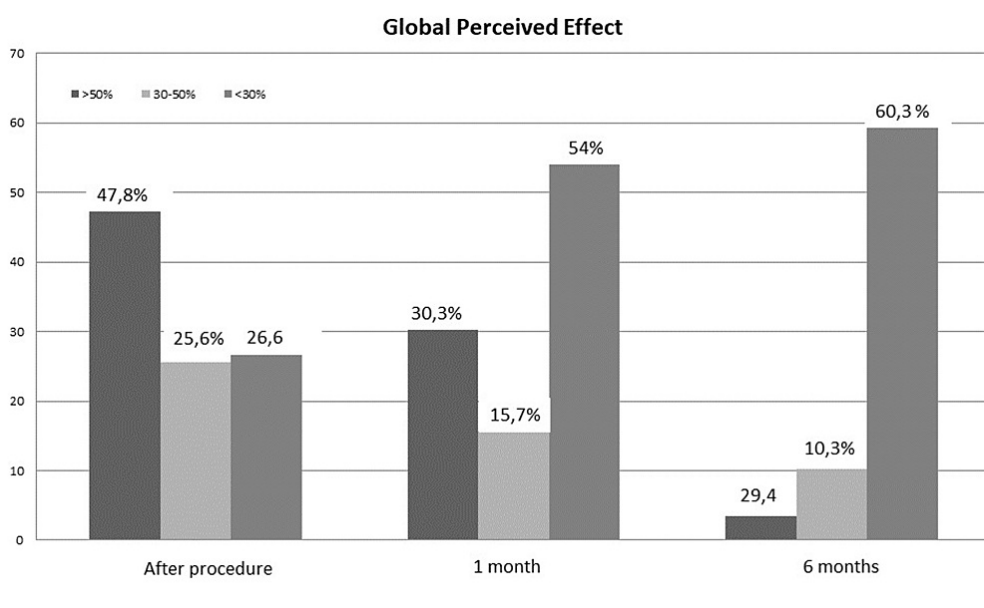
Global Perceived Effect

After the procedure, GPE was > 50% for 45 patients, 30-50% for 24 patients, and < 30% for 25 patients. At the one-month follow-up, GPE was > 50% for 27 patients, 30-50% for 14 patients, and < 30% for 48 patients. At 24 months, GPE was > 50% for 17 patients, 30-50% for six patients, and < 30% for 35 patients (Figure 2). At the 24-month follow-up, 28 patients started the process of spinal cord stimulator implantation due to insufficient analgesic coverage of periduroscopic adhesiolysis. Of these, two patients failed the trial, and eight complained of ineffectiveness, which led to the removal of the implant in four cases. Of the 28 patients who started the implantation procedure, 19 started with a post-periduroscopic GPE < 30%, five had 30-50%, and four had > 50%.

Cost evaluation of epiduroscopy vs. SCS

The total cost of periduroscopic adhesiolysis is € 2,472 per patient. The total cost of the SCS trial and final interventions is at least € 13,398 per patient. The costs for each component are shown in Table 2.

**Table 2:** Costs for procedure in Italian Health System

Cost element	Epiduroscopy	Spinal cord stimulation
Clinical preoperative evaluation (with ECG and blood exam)	79.2 €	79.2 €
Operating room	598 €	650 €
Personnel	76 €	300 €
Instruments	1.350 €	12.000 €
Imaging	19.5 €	19.5 €
Hospitalization	350 €	350 €
TOTAL	2.472 €	13.398 €

## Discussion

In the 106 patients analyzed, the periduroscopic adhesiolysis procedure was particularly effective immediately after the procedure and at the one-month follow-up. The pain relief showed a significant difference between baseline and immediately after the procedure, and this benefit persisted for the first month with a progressive return to the original level during the next 24 months (Figure 2). The GPE variation showed an excellent and modest outcome (> 50% and 30-50%) in 73.4% of patients immediately after the procedure and 58% at the one-month follow-up.

In the 24-month follow-up, the procedure tended to lose efficacy for patients, with optimal and intermediate outcomes occurring in 39.7%, as well as a change of 0.2 in NRS compared to the baseline. This result seems to agree with a recent review of the literature showing a progressively diminishing benefit over time [27]. Interestingly, the absence of pain reduction from baseline was not fully related to the GPE evaluation, showing how pain is not the only aspect that the patients consider in their outcomes. In the 58 patients analyzed at the 24-month follow-up, 28 (48%) started the procedure of SCS.

All 106 patients enrolled at the start of the study had potential indications for both SCS and epiduroscopy, but only 24 (41%) of the 58 patients analyzed at 24 months benefited from the SCS implant. The data seem to suggest that the periduroscopic adhesiolysis procedure tends to be an effective intermediate treatment to distinguish cases of pain mixed with a more significant inflammatory component, where epiduroscopy would be more effective, as well as cases of mixed pain with a more significant neuropathic component, where SCS is the only effective therapeutic option.

The cost per patient of the two procedures is significantly higher for SCS (€ 13,398/pt) than epiduroscopy (€ 2,472/pt) due to the high cost of the internal pulse generator (IPG) and device with the same length of hospitalization. Considering the 24-month follow-up with 58 patients, a therapeutic procedure that provides epiduroscopy plus the SCS implanted (€ 464,928) would be more economical than a procedure that provides for the preventive implantation of SCS for all patients without epiduroscopy attempt before (€ 770,084), with a total saving of € 305.106 (corresponding to € 5,261/pt).

The critical number of dropouts poses limitations to our study at 24 months and caused a substantial dispersion of data. We cannot say whether the dropout patients sought new surgical interventions or other medical therapy. As the primary center for the region, we can most likely affirm that those patients did not move to other pain centers for SCS implantation, but we cannot be completely sure. Further studies are needed to identify predictive factors that can predict which of the two methods will be more effective and to evaluate what other treatments will be effective after 24 months in patients who do not undergo spinal cord stimulation and have a lower NRS and GPE at baseline.

## Conclusions

The use of periduroscopic adhesiolysis as an intermediate procedure before SCS and can be included in the treatment regimen of severe lumbar pain that is refractory to pharmacological treatments and to reduce the use of SCS implants to save resources for the NHS. The analgesic relief of the periduroscopic procedure tends to lose efficacy after 24 months, but an increase in SCS implants does not accompany this trend. Further studies are needed to determine predictive factors that are capable of predicting the best treatment for the clinical needs of individual patients.
